# Original SARS-CoV-2 monovalent and Omicron BA.4/BA.5 bivalent COVID-19 mRNA vaccines: phase 2/3 trial interim results

**DOI:** 10.1038/s41591-023-02517-y

**Published:** 2023-08-31

**Authors:** Spyros Chalkias, Jordan L. Whatley, Frank Eder, Brandon Essink, Shishir Khetan, Paul Bradley, Adam Brosz, Nichole McGhee, Joanne E. Tomassini, Xing Chen, Xiaoping Zhao, Andrea Sutherland, Xiaoying Shen, Bethany Girard, Darin K. Edwards, Jing Feng, Honghong Zhou, Stephen Walsh, David C. Montefiori, Lindsey R. Baden, Jacqueline M. Miller, Rituparna Das

**Affiliations:** 1 Moderna, Inc., Cambridge, MA USA; 2 Meridian Clinical Research, Baton Rouge, LA USA; 3 Meridian Clinical Research, LLC, Binghamton, NY USA; 4 Meridian Clinical Research, Omaha, NE USA; 5 Meridian Clinical Research, Rockville, MD USA; 6 Meridian Clinical Research, Savannah, GA USA; 7 Meridian Clinical Research, Grand Island, NE USA; 8 Department of Surgery and Duke Human Vaccine Institute, Durham, NC USA; 9 Brigham and Women’s Hospital, Boston, MA USA

**Keywords:** Viral infection, Vaccines

## Abstract

This ongoing, open-label, phase 2/3 trial compared the safety and immunogenicity of the Omicron BA.4/BA.5-containing bivalent mRNA-1273.222 vaccine with the ancestral Wuhan-Hu-1 mRNA-1273 as booster doses. Two groups of adults who previously received mRNA-1273 as primary vaccination series and booster doses were enrolled in a sequential, nonrandomized manner and received single-second boosters of mRNA-1273 (*n* = 376) or bivalent mRNA-1273.222 (*n* = 511). Primary objectives were safety and the noninferiority or superiority of neutralizing antibody (nAb) responses against Omicron BA.4/BA.5 and ancestral SARS-CoV-2 with the D614G mutation (ancestral SARS-CoV-2 (D614G)), 28 days post boost. Superiority and noninferiority were based on prespecified success criteria (lower bounds of 95% CI > 1 and < 0.677, respectively) of the mRNA-1273.222:mRNA-1273 geometric mean ratios. Bivalent Omicron BA.4/BA.5-containing mRNA-1273.222 elicited superior nAb responses against BA.4/BA.5 versus mRNA-1273 and noninferior responses against ancestral SARS-CoV-2 (D614G) at day 29 post boost in participants without detectable prior SARS-CoV-2 infection. Day 29 seroresponses against Omicron BA.4/BA.5 were higher for mRNA-1273.222 than for mRNA-1273 and similar against ancestral SARS-CoV-2 (D614G), both meeting noninferiority criterion. The safety profile of mRNA-1273.222 was similar to that previously reported for mRNA-1273 with no new safety concerns identified. Continued monitoring of neutralization and real-world vaccine effectiveness are needed as additional divergent-virus variants emerge. ClinicalTrials.gov registration: NCT04927065.

## Abstract (web summary)

An Omicron BA.4/BA.5 mRNA booster vaccine elicits high neutralizing responses to the BA.4/BA.5 variant and to ancestral SARS-CoV-2, supporting tailoring booster vaccines to the predominant Omicron variant.

## Main

Booster immunization against severe acute respiratory syndrome coronavirus 2 (SARS-CoV-2) improves immune responses and vaccine effectiveness against coronavirus disease 2019 (COVID-19) (refs. ^1–8^). The emergence of antigenically divergent Omicron variants required variant-targeting immunization strategies and we previously evaluated the safety and immunogenicity of an Omicron BA.1-containing bivalent booster (mRNA-1273.214) (refs. ^5,9–12^). Neutralizing antibodies correlate with protection against many viral diseases^13^ including COVID-19 (refs. ^14,15^). The Omicron BA.1 bivalent vaccine increased the magnitude and the durability of the neutralizing antibody responses against BA.1 compared to mRNA-1273 (refs. ^5,6^), and exhibited cross-neutralization against Omicron BA.4/BA.5, BA.2.75, BQ.1.1 and XBB.1.1 variants. Results from a randomized study indicated improved protection against COVID-19 when updating the vaccine to more closely matched circulating variants^16^.

Due to the rapid SARS-CoV-2 evolution and the subsequent emergence of Omicron BA.4/BA.5 variants in spring and summer of 2022, a bivalent booster vaccine update containing the BA.4/BA.5 spike sequence (mRNA-1273.222) was also deployed to more closely match the dominant-circulating variant at the time. Given that the Omicron BA.4/BA.5 vaccine update had already been recommended as the standard-of-care^17^, a contemporaneous mRNA-1273 comparator group was not enrolled; instead a historical mRNA-1273 was used to compare the immune responses between mRNA-1273.222 and the original vaccine, mRNA-1273 (refs. ^18,19^).

Herein, we summarize the interim safety and immunogenicity results of the mRNA-1273.222 clinical study and we include neutralization information against variants that emerged after the Omicron BA.4/BA.5 wave. Rapid evaluation of variant-containing COVID-19 vaccines is important as vaccine updates are likely to be further recommended to enhance protection against COVID-19.

## Results

### Trial population

**Fig. 1:**
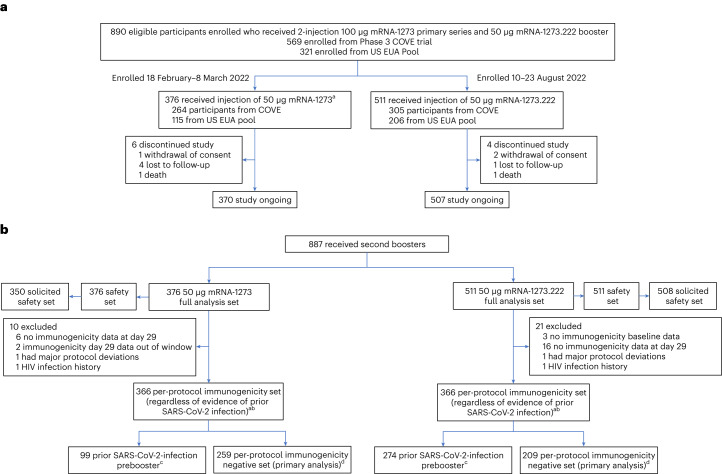
Enrollment and analysis populations in the trial. **a**, Trial profile. Eligible participants were sequentially enrolled as two cohorts to receive single-second booster doses of 50 µg mRNA-1273 (enrolled during 18 February–8 March 2022) or 50 µg mRNA-1273.222 (enrolled during 10–23 August 2022). ^a^379 participants were enrolled and received mRNA-1273; two participants had previously received the primary series but not a first booster dose and another participant had a major protocol deviation, and three were excluded from all analysis sets. Data cutoff dates were 23 September 2022 for mRNA-1273.222 at the day 29 interim analysis and 6 July 2022 for the within-study noncontemporaneous mRNA-1273 at the day 91 interim analysis^6^. **b**, Analysis populations. The full analysis set consisted of all participants who received study vaccine. The safety set consisted of all participants who received study vaccine and was used for all safety analyses except for solicited adverse reactions which were assessed in the solicited safety set. HIV, human immunodeficiency virus. ^a^The per-protocol set for immunogenicity consisted of all participants in the full analysis set who received the planned dose of study vaccination and had antibody data available at prebooster and day 29 and no major protocol deviations. ^b^Seven participants in the mRNA-1273.222 and eight participants in the mRNA-1273 arm of the per-protocol immunogenicity set had missing prebooster SARS-CoV-2 information. ^c^Prior SARS-CoV-2 infection based on positive RT–PCR and/or serology test at prebooster baseline. ^d^The per-protocol immunogenicity negative set (PPIS-negative) consists of participants in the per-protocol set for immunogenicity (PPIS) who have no serologic or virologic evidence of SARS-CoV-2 infection at prebooster baseline, that is, who are SARS-CoV-2 infection negative, based on both negative RT–PCR tests for SARS-CoV-2 and negative SARS-CoV-2 nucleocapsid antibody test, and is the primary analysis set for immunogenicity. A total of 379 participants received a second booster dose of 50 μg mRNA-1273; two participants had previously received the primary series but not a first booster dose, and another participant had a major protocol deviation. These three participants were excluded from all analysis sets. Source data

Between 10 and 23 August 2022, 511 participants received 50 µg of mRNA-1273.222. These participants had previously received the primary series of 100 µg mRNA-1273 and a first booster dose of 50 µg mRNA-1273, ≥3 months before enrollment (Fig. 1). Of these, 305 (59.7%) participants originated from the Coronavirus Efficiency (COVE) trial and 206 (40.3%) were US vaccinees under the emergency use authorization (EUA), respectively. Given that within the study enrollment windows the original booster vaccine mRNA-1273 was no longer authorized, we did not enroll a mRNA-1273 comparator group. Instead, we used a within-study, noncontemporaneous mRNA-1273 group (*n* = 379, enrollment 18 February–8 March 2022, data cutoff date of 6 July 2022 at the day 91 interim analysis)^5,6^ for the immunogenicity comparison. Four (0.8%) participants in the mRNA-1273.222 and six (1.6%) in the mRNA-1273 groups discontinued the study.

Participant demographics and baseline characteristics are shown in Table 1. Mean ages were 50.8 and 57.6 years, and 62% and 51% were female in the 50 µg mRNA-1273.222 and 50 µg mRNA-1273 groups, respectively. Most participants were White (83% and 86%), 11% and 7% were Black/African American and 11% and 10% were of Hispanic/Latinx ethnicity in the mRNA-1273.222 and mRNA-1273 groups, respectively. The percentages of participants with SARS-CoV-2 infection prebooster (positive binding antibody (bAb) against nucleocapsid protein^20^ or a positive polymerase chain reaction with reverse transcription (RT–PCR) test assessed at day 1) were 56% in the mRNA-1273.222 and 27% in the mRNA-1273 groups. The median times (days (interquartile range, IQR)) were similar between second doses of mRNA-1273 in the primary series and the first booster of mRNA-1273 for mRNA-1273.222 (251 (228–299)) and mRNA-1273 (242 (225–260)) groups, and were longer between the first mRNA-1273 booster and second booster doses in the mRNA-1273.222 (289 (258–312)) than in the mRNA-1273 (134 (118–150)) group.

**Table 1:** Demographics and study participant characteristics, safety set

Characteristics *n* (%)^a^	mRNA-1273.222 50 µg *N* = 511	mRNA-1273 50 µg *N* = 376
Age at screening (years)		
Mean (range)	50.8 (19–89)	57.6 (20–96)
Age subgroup		
≥18 and <65 years	406 (79.5)	226 (60.1)
≥65 years	105 (20.5)	150 (39.9)
Gender		
Male	195 (38.2)	186 (49.5)
Female	316 (61.8)	190 (50.5)
Ethnicity		
Hispanic or Latinx	58 (11.4)	37 (9.8)
Not Hispanic or Latinx	448 (87.7)	339 (90.2)
Not reported or unknown	5 (1.0)	0
Race		
White	426 (83.4)	322 (85.6)
Black or African American	56 (11.0)	28 (7.4)
Asian	11 (2.2)	16 (4.3)
American Indian or Alaska Native	1 (0.2)	1 (0.3)
Native Hawaiian or Other Pacific Islander	0	1 (0.3)
Multiracial	8 (1.6)	2 (0.5)
Other	6 (1.2)	2 (0.5)
Not reported or unknown	3 (0.6)	4 (1.1)
Time between second injection of mRNA-1273 in the primary series and the first booster of mRNA-1273 (days)		
*n*	509	374
Median	251.0	242.0
IQR	(228–299)	(225–260)
Time between first booster dose of mRNA-1273 and the second booster (days)		
* n*	509	374
Median	289.0	134.0
IQR	(258–312)	(118–150)
Prebooster RT–PCR SARS-CoV-2		
Negative	488 (95.5)	366 (97.3)
Positive	10 (2.0)	2 (0.5)
Missing	13 (2.5)	8 (2.1)
Prebooster antibody to SARS-CoV-2 nucleocapsid^b^		
Negative	226 (44.2)	276 (73.4)
Positive	282 (55.2)	100 (26.6)
Missing	3 (0.6)	0
Prebooster SARS-CoV-2 status^c^		
Negative	216 (42.3)	267 (71.0)
Positive	286 (56.0)	101 (26.9)
Positive by both RT–PCR and SARS-CoV-2 nucleocapsid^b^	6 (1.2)	1 (0.3)
Positive by RT–PCR only	4 (0.8)	1 (0.3)
Positive by SARS-CoV-2 nucleocapsid only^b^	276 (54.0)	99 (26.3)
Missing	9 (1.8)	8 (2.1)

^a^Percentages based on the number of participants in the safety set.

^b^Elecsys assay for binding antibody to SARS-CoV-2 nucleocapsid.

^c^Prebooster SARS-CoV-2 status was positive if there was evidence of prior SARS-CoV-2 infection, defined as positive binding antibody against the SARS-CoV-2 nucleocapsid or positive RT–PCR at day 1; negative SARS-CoV-2 status was defined as negative binding antibody against the SARS-CoV-2 nucleocapsid and a negative RT–PCR at day 1.

### Safety

**Fig. 2:**
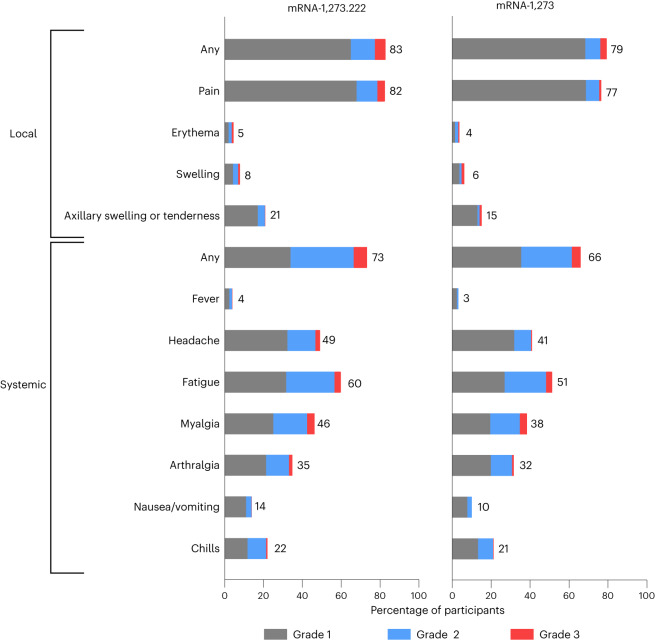
Solicited local and systemic adverse reactions. Percentages of participants who had solicited local or systemic adverse reactions within 7 days by grade after the 50 µg mRNA-1273.222 second booster dose (left) and mRNA-1273 historical group (right) at day 91 (ref. ^6^) in the solicited safety sets.

The overall safety profile of the mRNA-1273.222 booster is similar to that of the previously described 50 µg mRNA-1273 historical group at 28 days post booster dose^5,6^. Median durations of follow-up days (IQR) were 37.0 (33.0–39.0) for the mRNA-1273.222 participants and 127 (125–132) for the historical mRNA-1273 group^6^. Previously reported solicited local and systemic adverse reactions within 7 days after injection for mRNA-1273 are shown in Fig. 2 for comparison with those of mRNA-1273.222. The most frequently reported local adverse reaction after administration of mRNA-1273.222 was injection-site pain. The most frequent systemic reactions were fatigue, headache, myalgia and arthralgia (Fig. 2 and Supplementary Table 4). The majority of solicited adverse reactions were mild-to-moderate (grades 1–2) and the most common grade 3 reactions were fatigue and myalgia; no grade 4 reactions occurred. Median duration days (IQR) were 3 (2–4) for local and 3 (1–5) for systemic adverse reactions and persistence beyond 7 days occurred in 1.6% of participants for local reactions and 7.7% for systemic reactions (Supplementary Table 5). Grade 3 events of erythema (1 (0.2%)), headache (1 (0.2%)) and fatigue (4 (0.8%)) persisted beyond 7 days. The frequency of local and systemic adverse reactions was 84.7% and 77.3%, respectively, in participants without prior SARS-CoV-2 infection and 81.2% and 69.6% in those with prior SARS-CoV-2 infection.

The incidence of unsolicited adverse events regardless of the relationship to vaccination ≤28 days after the booster dose of mRNA-1273.222 was 23% (Supplementary Table 6) and similar to that reported previously for mRNA-1273 (ref. ^6^); the incidence of unsolicited adverse events considered to be related to study vaccination by the investigator was 8%. Three (0.6%) participants in the mRNA-1273.222 group experienced serious adverse events (anginal equivalent and syncope, anemia, fatal event of subarachnoid hemorrhage); none were considered related to study vaccination by the investigators. Medically attended adverse events occurred in 14% of mRNA-1273.222 participants; none were considered related to study vaccination by investigators. Two grade 3 events of fatigue, considered related to study vaccination, were reported. No events of myocarditis or pericarditis and no adverse events leading to study discontinuation occurred in this interim analysis.

### Immunogenicity

**Fig. 3:**
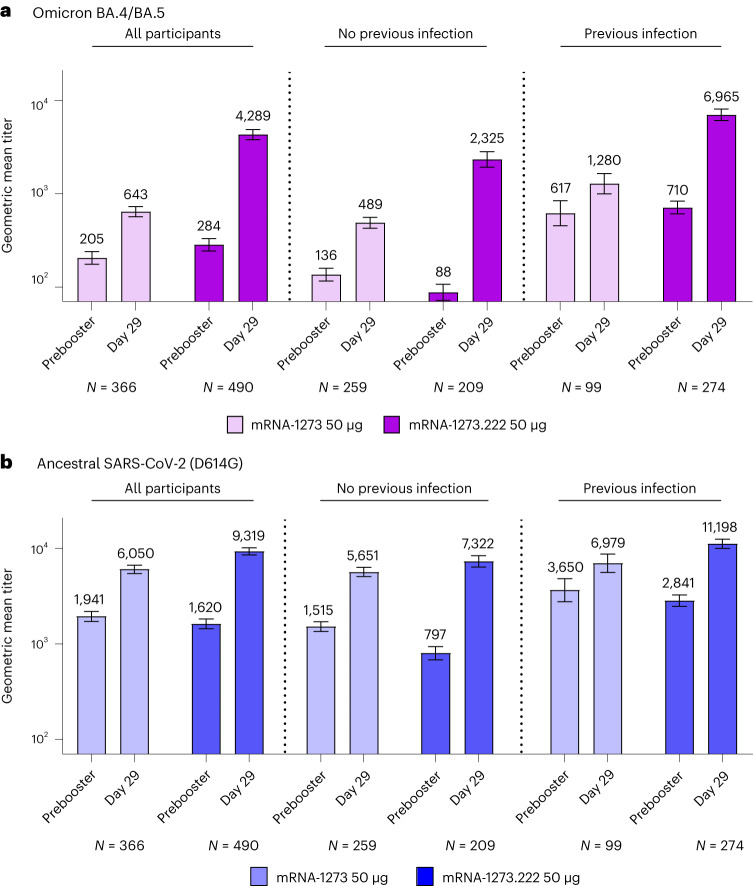
nAb titers after 50 µg of mRNA-1273.222 and mRNA-1273 administered as second booster doses. **a**,**b**, Observed nAb titers against Omicron BA.4/BA.5 (**a**) and ancestral SARS-CoV-2 (D614G) (**b**) after 50 µg of mRNA-1273.222 and mRNA-1273 administered as second booster doses. Pseudovirus nAb GMTs are provided for all participants regardless of SARS-CoV-2 infection prebooster status, and those with and without previous SARS-CoV-2 infection prebooster. Data are from participants with nonmissing data at the timepoint. Seven participants in mRNA-1273.222 and eight participants in the mRNA-1273 group were missing prebooster SARS-CoV-2 status in the per protocol immunogenicity set. Antibody values reported as below the LLOQ (18.5 (1.3 in log_10_ scale) for ancestral SARS-CoV-2 (D614G) and 36.7 (1.6 in log_10_ scale) for Omicron BA.4/BA.5) were replaced by 0.5 × LLOQ. Values greater than the upper limit of quantification (ULOQ 45,118 (4.7 in log_10_ scale) for ancestral SARS-CoV-2 (D614G); 13,705 (4.1 in log_10_ scale) for Omicron BA.4/BA.5) were replaced by the ULOQ if actual values were not available. 95% CIs were calculated based on the *t*-distribution of the log-transformed values for GM value, then back-transformed to the original scale for presentation. Data for observed nAb GMTs by prior SARS-CoV-2 infection are provided in Supplementary Table 7.

**Extended Data Fig. 1:**
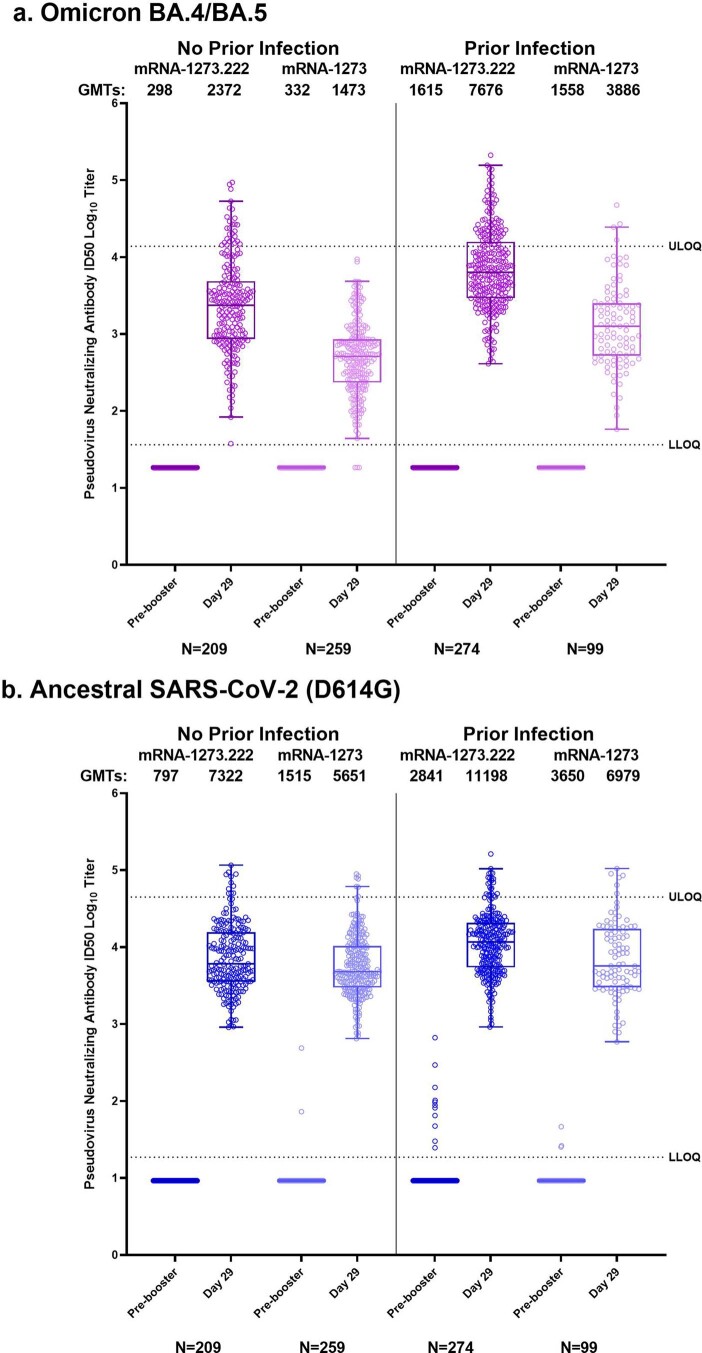
Distribution of Observed Neutralizing Antibody Titers. Distribution of neutralizing antibody titers (ID50 log_10_) in the pseudovirus assay against Omicron BA.4/BA.5 (Panel A) and ancestral SARS-CoV-2 (D614G) (Panel B) are shown for serum samples collected before the second booster dose of 50-µg of mRNA-1273.222 or 50-µg of mRNA-1273 (prebooster), and at 28 days after the second booster dose (day 29) in those with and without prior SARS-CoV-2 infection. The circles are values from individual serum samples. Pseudovirus neutralizing antibody assay lower limits of quantification (LLOQ) are 18.5 (1.3 log_10_) for ancestral SARS-CoV-2 [D614G] and 36.7 (1.6 log_10_) for Omicron BA.4/BA.5; upper limits of quantification (ULOQ) are 45,118 (4.7 log_10_) for ancestral SARS-CoV-2 [D614G] and 13,705 (4.1 log_10_) for Omicron BA.4/BA.5. Boxes and horizontal bars denote interquartile (IQR) ranges and median endpoint titers; whisker endpoints are the maximum and minimum values below or above the median ±1.5 times the IQR. Refer to Table 2 for the observed geometric mean titers and geometric mean fold-rises, and the estimated neutralizing antibody geometric mean titers and geometric mean ratios (ANCOVA model-based) following the second booster doses of 50-µg mRNA-1273 or 50-µg of mRNA-1273.214.

**Extended Data Fig. 2:**
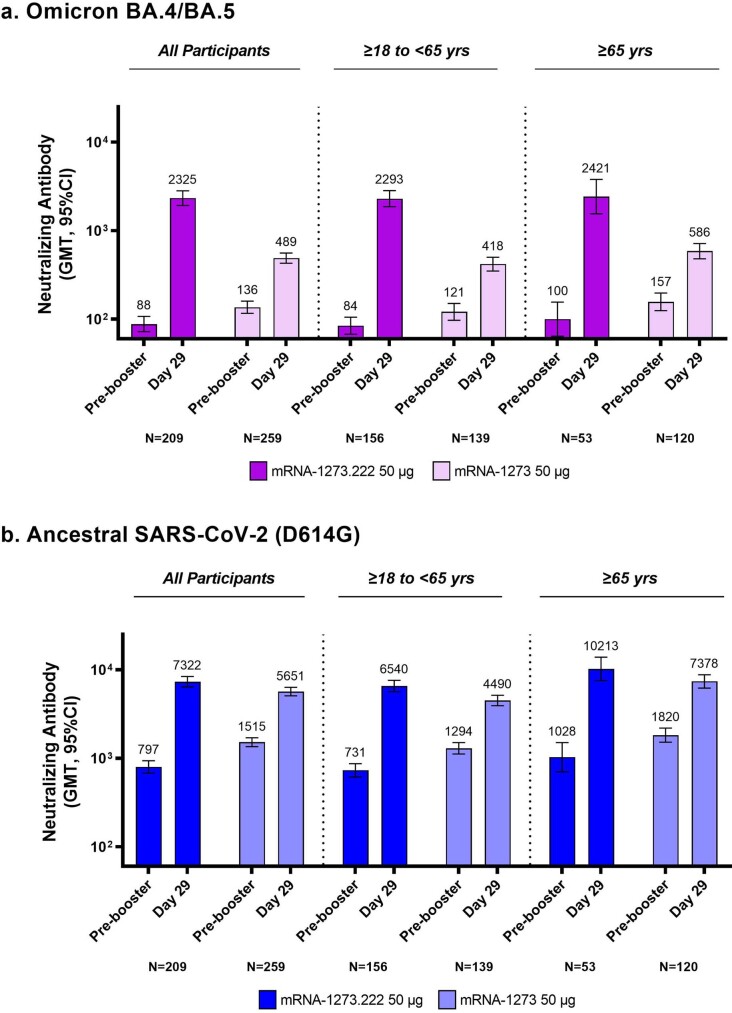
Observed Neutralizing Antibody Response by Age Groups. Observed neutralizing antibody titers (GM titer log10) in the pseudovirus assay against Omicron BA.4/BA.5 (a) and ancestral SARS-CoV-2 (D614G) (b) are shown for serum samples collected before the second booster dose of 50-µg of mRNA-1273.222 or 50-µg of mRNA-1273 (prebooster) (day 0), and at 28 days (day 29) after the second booster dose in all participants and those ≥18 to <65 or ≥65 years of age in the per-protocol immunogenicity set without evidence of prebooster SARS-CoV-2 infection. N=Number of participants with nonmissing data at the timepoint; CI=Confidence interval; GMT=geometric mean titer; LLOQ=lower limit of quantification; ULOQ=upper limit of quantification; PPIS=Per-protocol immunogenicity set. Antibody values assessed by pseudovirus neutralizing antibody assay reported as below the LLOQ (18.5 [1.3 in log10 scale] for ancestral SARS-CoV-2 (D614G) and 36.7 [1.6 in log10 scale] for Omicron BA.4/BA.5) are replaced by 0.5 × LLOQ. Values greater than ULOQ (45,118 [4.7 in log10 scale] for ancestral SARS-CoV-2 (D614G) and 13,705 [4.1 in log10 scale] for Omicron BA.4/BA.5) are replaced by the ULOQ if actual values are not available. Includes participants in the per-protocol immunogenicity set without evidence of prebooster SARS-CoV-2 infection (PPIS-negative). 95% CIs were calculated based on the t-distribution of the log-transformed values for GM value, then back-transformed to the original scale for presentation.

In participants without evidence of prior SARS-CoV-2 infection 28 days after the mRNA-1273.222 and mRNA-1273 booster dose, respectively, the observed neutralizing antibodies (nAb), reported as geometric mean titers (GMTs (95% CI)), were 2,324.6 (1,921.2−2,812.7) and 488.5 (427.4−558.4) against Omicron BA.4/BA.5, and 7,322.4 (6,386.2−8,395.7) and 5,651.4 (5,055.7−6,317.3) against ancestral SARS-CoV-2 (D614G) (Fig. 3, Extended Data Fig. 1 and Table 2). Analysis of covariance (ANCOVA)-estimated GMTs after adjusting for age groups and prebooster titers 28 days after the mRNA-1273.222 and mRNA-1273 booster dose, respectively, were 2,747.3 (2,399.2−3,145.9) and 436.7 (389.1−490.0) against Omicron BA.4/BA.5 and 9,555.8 (8,593.6−10,625.7) and 4,882.2 (4,457.7−5,347.1) against ancestral SARS-CoV-2 (D614G). The geometric mean ratio (GMR (95% CI)) against Omicron BA.4/BA.5 was 6.29 (5.27−7.51), meeting the prespecified superiority criterion (lower bound of the GMR 95% CI > 1). The GMR (95% CI) against ancestral SARS-CoV-2 (D614G) was 1.96 (1.70−2.25), meeting the prespecified criterion for noninferiority. Seroresponse rates (SRR) (change from < lower limit of quantification (LLOQ) to ≥4 × LLOQ, or at least a fourfold rise if baseline ≥LLOQ) compared with the preinjection 1 baseline (95% CI) against Omicron BA.4/BA.5 were 98.1% (95.2−99.5%) for mRNA-1273.222 and 86.4% (81.6−90.3%) for mRNA-1273, and SRR against ancestral SARS-CoV-2 (D614G) were 100% (98.3−100% and 98.6−100%) 28 days after the mRNA-1273.222 and mRNA-1273 booster doses, respectively, with estimated differences of 12.1 (6.9−17.3) and 0, both meeting the prespecified noninferiority criterion. Therefore, all primary and immunogenicity objectives were met (Supplementary Fig. 1). Neutralizing titers against Omicron BA.4/BA.5 and ancestral SARS-CoV-2 (D614G) were also consistently higher with mRNA-1273.222 than with mRNA-1273 at day 29 among those ≥65 years and 18–<65 years of age (Extended Data Fig. 2). In participants with evidence of prior SARS-CoV-2 infection, GMTs were higher following the mRNA-1273.222 than mRNA-1273 booster against both Omicron BA.4/BA.5 and ancestral SARS-CoV-2 (D614G) with GMRs (95% CI) of 5.11 (4.10−6.36) and 1.84 (1.56−2.18), respectively (Fig. 3 and Supplementary Table 7).

**Table 2:** Primary immunogenicity analysis of Omicron BA.4/BA.5 and ancestral SARS-CoV-2 (D614G) after 50 µg of mRNA-1273.222 and mRNA-1273 administered as second booster doses in participants with no prior SARS-CoV-2 infection

	Ancestral SARS-CoV-2 (D614G)		Omicron BA.4/BA.5	
	50 µg mRNA-1273.222 booster dose	50 μg mRNA-1273 booster dose	50 µg mRNA-1273.222 booster dose	50 μg mRNA-1273 booster dose
	*N* = 209	*N* = 259	*N* = 209	*N* = 259
Prebooster *n*^a^	209	259	209	259
Observed GMT (95% CI)^b^	796.9 (678.7–935.8)	1,515.4 (1347.5–1704.2)	87.9 (72.2–107.1)	136.1 (116.3–159.3)
Day 29, *n*^a^	209	259	209	259
Observed GMT (95% CI)^b^	7,322.4 (6,386.2–8,395.7)	5,651.4 (5055.7–6317.3)	2,324.6 (1,921.2–2,812.7)	488.5 (427.4–558.4)
GMFR (95% CI)^b^	9.2 (7.9–10.6)	3.7 (3.4–4.1)	26.4 (22.0–31.9)	3.6 (3.3–4.0)
Estimated GMT (95% CI)^c^	9,555.8 (8,593.6–10,625.7)	4,882.2 (4,457.7–5,347.1)	2,747.3 (2,399.2–3,145.9)	436.7 (389.1–490.0)
GMR (95% CI)^c,d^	1.96 (1.70–2.25)		6.29 (5.27–7.51)^e^	
Day 29 SRR, injection 1, *n*/N1, %^f^	209/209, 100	259/259, 100	205/209, 98.1	222/257, 86.4
(95% CI)	(98.3–100.0)	(98.6–100.0)	(95.2–99.5)	(81.6–90.3)
Difference (95% CI)^d,g^	0		12.1 (6.9–17.3)	
Day 29 SRR, prebooster, *n*/N1, %^f^	168/209, 80.4	111/259, 42.9	190/209, 90.9	98/259, 37.8
(95% CI)	(74.3–85.5)	(36.7–49.1)	(86.2–94.4)	(31.9–44.0)
Difference (95% CI)^d,g^	37.3 (29.0–45.6)		53.9 (46.7–61.2)	

Antibody values assessed by pseudovirus neutralizing antibody assay reported as below the LLOQ (18.5 for ancestral SARS-CoV-2 (D614G) and 36.7 for Omicron BA.4/BA.5) are replaced by 0.5 × LLOQ. Values greater than ULOQ (45,118 for ancestral SARS-CoV-2 (D614G) and 13,705 for Omicron BA.4/BA.5) are replaced by the ULOQ if actual values are not available. Includes participants with no prior SARS-CoV-2 infection, primary analysis set.

N1 = Number of participants with nonmissing data at baseline (prebooster) and the corresponding post baseline timepoint.

^a^Number of participants with nonmissing data at the timepoint (prebooster baseline or post baseline).

^b^GMT, GM FR of nAb at day 29 post baseline timepoint over predose day 1 with corresponding 95% CI based on the *t*-distribution of log-transformed values or difference in the log-transformed values for GMT value and GMT fold-rise, respectively, then back-transformed to the original scale.

^c^Log-transformed antibody levels are analyzed using an ANCOVA model with the treatment variable as fixed effect, adjusting for age group (<65, ≥65 years) and prebooster titers. The resulting least squares (LS) means, difference of LS means and 95% CI are back-transformed to the original scale.

^d^95% CI was calculated by stratified Miettinen–Nurminen method adjusted by age group.

^e^Exceeded noninferiority criteria and met superiority criteria including lower bound CI > 1 and testing sequence.

^f^Seroresponse at a participant level defined as a change from <LLOQ to ≥4 × LLOQ, or at least a fourfold rise if baseline (preinjection 1/prebooster is ≥LLOQ; comparison to preinjection 1/prebooster baselines as indicated. Percentages were based on the number of participants with nonmissing data at baseline and the corresponding timepoint, 95% CI was calculated using the Clopper–Pearson method.

^g^The SRR difference is a calculated common risk difference using inverse-variance stratum weights and the middle point of Miettinen–Nurminen confidence limits of each one of the stratum risk differences. The stratified Miettinen–Nurminen estimate and the CI cannot be calculated when the SRR in both groups is 100%, absolute difference is reported.

**Extended Data Fig. 3:**
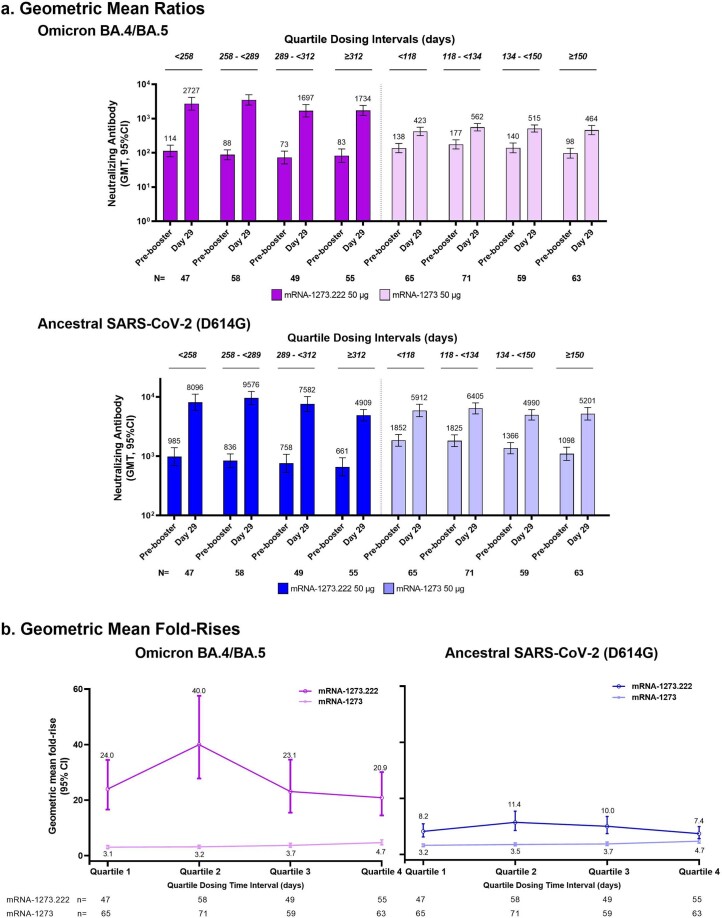
Neutralizing Antibody Titers and Geometric Fold-Rises by Time Intervals. Neutralizing antibody titers against Omicron BA.4/BA.5 and ancestral SARS-CoV-2 (a) and geometric fold-rises from baseline levels (b) were assessed in participants without previous infection grouped by quartiles of dosing intervals between the first booster dose of mRNA-1273 and second booster doses of mRNA-1273 and mRNA-1273.222 post boost within each booster vaccine group at day 29. 95% CIs were calculated based on the t-distribution of the log-transformed values for GM value, then back-transformed to the original scale for presentation. Quartiles 1, 2, 3 and 4 represent quartiles indicated s in panel a for mRNA-1273 and mRNA-1273.222.

To explore whether time intervals between the first and second booster doses influenced the post booster nAb GMTs, antibody responses against Omicron BA.4/BA.5 and ancestral SARS-CoV-2 (D614G) across quartile time intervals for the mRNA-1273.222 and mRNA-1273 groups were analyzed among participants with no previous infection (Extended Data Fig. 3 and Supplementary Table 8). Antibody titers did not appear to increase as the interval between prior doses increased within each treatment group. The GMTs and geometric mean fold-rise (GMFR) for mRNA-1273.222 increased at the day 258–289 interval, then decreased over time to ≥312 days against both Omicron BA.4/BA.5 and ancestral SARS-CoV-2 (D614G). The GMTs and GMFR of mRNA-1273 remained generally consistent as the intervals increased.

**Fig. 4:**
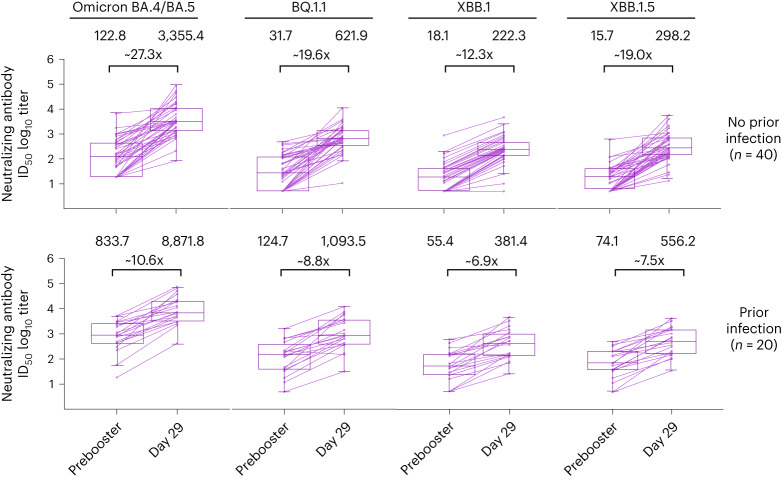
nAb titers against Omicron variants after 50 µg of mRNA-1273.222 administered as a second booster dose by prior SARS-CoV-2 infection status at prebooster. Prebooster and day 29 nAb titers (log_10_) against Omicron BA.4/BA.5, BQ.1.1, XBB.1 and XBB.1.5 variants in randomly selected subsets of participants in the mRNA-1273.222 booster group with (*n* = 20) and without (*n* = 40) prior SARS-CoV-2 infection. Prebooster and day 29 geometric mean titers and geometric mean fold-rises from baseline (X) are displayed above the corresponding data points. Antibody values reported as below the LLOQ (18.5 (1.3 in log_10_ scale) for ancestral SARS-CoV-2 (D614G) and 36.7 (1.6 in log_10_ scale) for Omicron BA.4/BA.5) were replaced by 0.5 × LLOQ. Values greater than the ULOQ (45,118 (4.7 in log_10_ scale) for ancestral SARS-CoV-2 (D614G); 13,705 (4.1 in log_10_ scale) for Omicron BA.4/BA.5) were replaced by the ULOQ if actual values were not available. The limit of detection (LOD) of the pseudovirus nAb assay for BQ.1.1, XBB.1 and XBB.1.5 was 10; antibody values reported as below the LOD are replaced by 0.5 × LOD. Boxes and horizontal bars denote IQR and median endpoint titers; whisker endpoints are the maximum and minimum values below or above the median ±1.5 times the IQR.

Lastly, binding antibody (bAb, reported as geometric mean (GM) levels) GM levels across Omicron BA.1, the ancestral SARS-CoV-2, Alpha, Beta, Gamma and Delta variants were also higher following mRNA-1273.222 than mRNA-1273 boosters, and GMRs (95% CI) ranged from 1.75 (1.57−1.96) to 2.03 (1.81−2.27) in participants with no prior infection (Supplementary Table 9), and GMRs (95% CI) ranged from 1.52 (1.35−1.71) to 1.79 (1.58−2.03) in participants with prior infection (Supplementary Table 10).

Given the emergence of the Omicron BQ.1.1, XBB.1 and XBB.1.5 sublineages with the potential for immune escape, neutralization at day 29 against these variants was assessed in exploratory analyses for mRNA-1273.222 as described in the Methods (Fig. 4 and Supplementary Table 11). In a randomly selected subset of mRNA-1273.222 participants without prior detectable SARS-CoV-2 infection (*n* = 40), prebooster GMTs (95% CI) against BA.4/5, BQ.1.1, XBB.1 and XBB.1.5, respectively, were 122.8 (74.3−203.1), 31.7 (19.6−51.3), 18.1 (12.0−27.1) and 15.7 (10.7–23.1); day 29 post booster GMTs were 3,355.4 (2,109.9−5,336.2), 621.9 (422.2−916.2), 222.3 (147.4−335.2) and 298.2 (196.8–451.8) with fold-rises (95% CI) from prebooster titers of 27.3 (15.9−47.0), 19.6 (11.7−32.8), 12.3 (7.4−20.5) and 19.0 (11.9–30.2). Neutralizing BQ.1.1, XBB.1 and XBB.1.5 titers were 5.4-, 15.1- and 11.3-fold lower than the corresponding BA.4/BA.5 titers. In mRNA-1273.222 participants with prior infection (*n* = 20), prebooster GMTs against BA.4/5, BQ.1.1, XBB.1 and XBB.1.5, respectively, were 833.7 (422.5−1645.1), 124.7 (61.4−253.2), 55.4 (28.4−108.0) and 74.1 (38.6–142.6) and the day 29 post booster GMTs were 8,871.8 (4,809.7−16,364.8), 1,093.5 (536.8−2,227.9), 381.4 (198.1−734.4) and 556.2 (299.1–1,034.4), with fold-rises of 10.6 (6.4−17.6), 8.8 (5.0−15.5), 6.9 (4.0−11.7) and 7.5 (4.5–12.6) from prebooster titers. The BQ.1.1, XBB.1 and XBB.1.5 nAb titers were 8.1-, 23.3- and 16.0-fold lower than the corresponding BA.4/BA.5 titers.

### Incidence of SARS-CoV-2 infections

In mRNA-1273.222 participants starting 14 days post booster regardless of prebooster SARS-CoV-2 infection status, 17 (3.3%) SARS-CoV-2 infections occurred with nine (1.8%) asymptomatic infections and eight (1.6%) symptomatic COVID-19 events, per the CDC definition, and were comparable to incidences previously reported for 50 µg mRNA-1273 as well as for an Omicron BA.1 bivalent booster vaccine (mRNA-1273.214) (Supplementary Table 12)^5,6^. There were no emergency room visits or hospitalizations due to these COVID-19 events.

## Discussion

The rapid evolution of antigenically divergent SARS-CoV-2 variants precipitated development of booster immunizations to maintain protection against COVID-19 and regulatory agencies have authorized variant-containing bivalent booster vaccines globally^9–11,21^. Given the pace of viral evolution and need for agility, regulatory agencies authorized Omicron BA.4/BA.5 bivalent boosters based on BA.1 bivalent vaccine clinical trial data^5,6^ and BA.4/BA.5 bivalent vaccine animal study information available at the time. The present study provides key clinical safety and immunogenicity data which support authorization of the Omicron BA.4/BA.5 bivalent booster mRNA-1273.222. The rapid manufacturing and deployment of this vaccine, within months, is in response to the speed with which SARS-CoV-2 variants of consequence (BA.4/BA.5) spread and demonstrates that flexible vaccine platforms, such as mRNA, are an important tool as divergent SARS-CoV-2 variants continue to emerge.

The 50 µg bivalent vaccine mRNA-1273.222 elicited higher BA.4/BA.5 neutralizing antibody responses compared to the original 50 µg mRNA-1273 28 days after the booster dose, in participants with and without evidence of SARS-CoV-2 infection on the day of the booster dose. Additionally, mRNA-1273.222 exhibited cross-neutralization against divergent variants which are not contained in the vaccine, such as BQ.1.1, XBB.1 and XBB.1.5, although the antibody titers for these variants were lower compared to BA.4/BA.5. These results are consistent with cross-neutralization observed after the BA.1-Omicron-containing mRNA-1273.214 bivalent vaccine dose as titers against Omicron BA.4/BA.5, BQ.1.1 and XBB.1 were lower compared to the matched BA.1 variant in the mRNA-1273.214 vaccine^6^. Results from a randomized, active-controlled clinical trial comparing an Omicron BA.1-containing booster vaccine with mRNA-1273, suggest a trend towards improved vaccine efficacy with the variant-containing booster, particularly when the variant is more closely antigenically matched to the variant in the vaccine^16^. The mechanism of immune responses elicited by bivalent boosters is yet to be elucidated. Studies suggest that immunization with Omicron-containing vaccines elicits new germinal centers and de novo B cell populations that likely contribute to enhanced neutralization, and that pre-existing T cell immune responses also contribute to immune responses to SARS-CoV-2 variants^7,22^.

Our interim results also indicate that the incidence of adverse reactions with the Omicron BA.4/BA.5-containing bivalent booster mRNA-1273.222 was similar to that of prior doses of mRNA-1273 (refs. ^1,23,24^), with no increased reactogenicity in participants with evidence of SARS-CoV-2 infection at prebooster. The mRNA-1273.222 safety information together with the previously reported 3-month safety follow-up on the BA.1 bivalent booster mRNA-1273.214 (refs. ^5,6^), add to the evaluation of the safety profile of Omicron-targeting bivalent vaccines which appear to be similar to that of the original mRNA-1273 vaccine, and suggest that safety is consistent for mRNA-1273 vaccines regardless of the bivalent variant modification. Longer-term evaluation of the safety of mRNA-1273.222 continues in this ongoing study.

Limitations include that the study was not randomized. Given that the Omicron BA.4/BA.5 vaccine update had already been recommended as the standard-of-care^17^, a contemporaneous mRNA-1273 comparator group was not enrolled; instead a historical mRNA-1273 was used to compare the immune responses between mRNA-1273.222 and mRNA-1273 (ref. ^18^). Although under such circumstances, use of a historical comparator evaluated using the same immunogenicity assay is advised by the regulatory guidance^19^, differences between the two vaccine groups limit the interpretation of our results despite model-adjusting the neutralizing antibody responses to account for potential confounding factors. The different intervals between prior booster doses and the likelihood that prebooster infections were caused by different circulating variants during the enrollment times between the two groups (Supplementary Fig. 2) could have a distinct impact on the development of immune responses in each group. We used the antinucleocapsid antibody test to assess previous SARS-CoV-2 infection and although a vaccine-induced reduction in seroconversion is possible when using this test^25,26^, the distinction between uninfected and infected persons is becoming less relevant given that the majority of the global population has been infected with SARS-CoV-2, with seroprevalence increases from 16% in February 2021 to 67% by October 2021 (ref. ^27^). Overall seroprevalence rates in high-income countries through March 2022 were 95.9% (92.3–97.8%) in Europe and 99.8% (99.7–99.9%) in the Americas from 4.3% (3.4–5.5%) and 3.6% (2.5–5.2%), respectively in June 2020 (ref. ^28^). mRNA-1273.222 antibody responses against BQ.1.1, XBB.1 and XBB.1.5 were assessed in subgroups and at only day 29 post booster to enable rapid evaluation, and responses were not assessed for mRNA-1273 as use of this booster has been withdrawn. XBB lineage variants subsequent to XBB.1 and XBB.1.5 (for example XBB.1.16) are antigenically similar to XBB.1.5 and neutralization was not assessed separately^6,29,30^. Lastly, this study was not designed to evaluate vaccine effectiveness and while the low number of COVID-19 events post boost precludes an interpretation of efficacy, the incidences are similar to those previously reported for Omicron BA.1 bivalent and original vaccine groups (Supplementary Table 12)^5,6^, and to randomized relative vaccine efficacy trials as well as real-world vaccine effectiveness data^8,16,31–34^.

In conclusion, given that SAS-CoV-2 variants are most likely to continue to emerge, our study provides a model for rapid clinical evaluation of a COVID-19 vaccine when a booster update and deployment is needed. Our results indicate that the Omicron BA.4/BA.5 bivalent mRNA-1273.222 vaccine increased neutralizing antibody responses against Omicron variants, with no new safety concerns compared to the original vaccine mRNA-1273. Continuous monitoring of cross-neutralization and vaccine effectiveness are needed to ensure updated vaccination strategies against COVID-19.

## Methods

### Study design

This is an open-label, ongoing, phase 2/3 study to evaluate the immunogenicity, safety and reactogenicity of various modified mRNA-1273 vaccine candidates against COVID-19 administered as boosters (mRNA-1273.211 (Part A.1), mRNA-1273 (Part B), mRNA-1273.617.2 (Part C), mRNA-1273.213 (Parts D and E), mRNA-1273.529 (Part F), mRNA-123.214 (Part G and Part A.2) and mRNA-1273.222 (Part H) vaccines; clintrials.gov NCT04927065). Part H interim results are reported here. Part H evaluates the safety, reactogenicity and immunogenicity of the bivalent Omicron BA.4/BA.5-containing mRNA-1273.222 (25 µg each of two mRNAs encoding the ancestral SARS-CoV-2 (Wuhan) and Omicron variant (BA.4/BA.5) spike sequences) booster vaccine compared to the currently authorized mRNA-1273 booster in adults who had previously received a two-injection primary series (100 µg) and first booster doses (50 µg) of mRNA-1273 in the COVE trial^23,24^ or under US EUA. The noncontemporaneous mRNA-1273 group^5,6^ serves as a within-study comparator for the prespecified immunogenicity comparison between mRNA-1273.222 and mRNA-1273 given that enrollment with the original vaccine mRNA-1273 was no longer feasible after the US FDA recommendation and authorization of bivalent booster vaccines^18,19,21^.

Participants were enrolled in a sequential, nonrandomized manner and received single-second boosters of the within-study noncontemporaneous 50 µg mRNA-1273 (enrolled between 18 February and 8 March 2022) or 50 µg bivalent mRNA-1273.222 (enrolled between 10 August and 23 August 2022). Data are reported for the mRNA-1273 group (Part F, cohort 2) based on the data cutoff date of 6 July 2022 at the day 91 interim analysis^6^, and for mRNA-1273.222 (Part H) based on the 29-day interim analysis data cutoff date of 23 September 2022.

### Trial registration

The study protocol was approved by the Institutional Review Board on 26 May 2021, and enrollment in part A of the study was initiated on 28 May 2021, before the trial posting date of 14 June 2021 on the clintrials.gov registration website as we were emergently advancing COVID-19 booster vaccine candidates to combat the SARS-CoV-2 pandemic (the objective of this trial). Nonetheless, the trial was registered within 21 days after the first participant was enrolled, compliant per clinicaltrials.gov guidance (https://clinicaltrials.gov/ct2/manage-recs/fdaaa). Additionally, in parts F and H of the study presented in this manuscript, the 890 participants were enrolled during 10–23 August 2022 for mRNA-1273.222 (Part H) and during 18 February–8 March 2022 (Part F, cohort 2) for mRNA-1273. Moderna received EUA and/or approvals of booster vaccine mRNA-1273 50 µg and bivalent booster candidates mRNA-1273.214 by regulatory agencies^17^ as well as for mRNA-1273.222 presented in this part (H) of the trial^18^.

The trial is being conducted across 23 US sites, in accordance with the International Council for Harmonisation of Technical Requirements for Registration of Pharmaceuticals for Human Use, Good Clinical Practice guidelines. The central Institutional Review Board (Advarra, Inc., 6100 Merriweather Drive, Columbia, MD 21044) approved the protocol and consent forms. All participants provided written informed consent. The study was funded by the sponsor, Moderna, Inc. and was involved in the study design as well as the collection, analysis and interpretation of the data.

### Participants

Adults with a known history of SARS-CoV-2 infection ≤3 months from screening were excluded. Additional inclusion/exclusion criteria specific to Part H participant eligibility include:

#### Inclusion criteria

Each participant must meet all of the following criteria to be enrolled in this study:

1. Male or female, at least 18 years of age at the time of consent (Screening Visit).
2. Investigator’s assessment that participant understands and is willing and physically able to comply with protocol-mandated follow-up, including all procedures.
3. Participant has provided written informed consent for participation in this study, including all evaluations and procedures as specified in this protocol.
4. Female participants of nonchildbearing potential may be enrolled in the study.
  Nonchildbearing potential is defined as surgically sterile (history of bilateral tubal ligation, bilateral oophorectomy, hysterectomy) or postmenopausal (defined as amenorrhea for ≥12 consecutive months before Screening (day 0) without an alternative medical cause). A follicle-stimulating hormone level may be measured at the discretion of the investigator to confirm postmenopausal status.
5. Female participants of childbearing potential may be enrolled in the study if the participant fulfills all of the following criteria:
  Has a negative pregnancy test on the day of vaccination (day 1)
  Has practiced adequate contraception or has abstained from all activities that could result in pregnancy for at least 28 days before day 1
  Has agreed to continue adequate contraception through 3 months following vaccination
  Is not currently breastfeeding
  (Adequate female contraception is defined as consistent and correct use of a Food and Drug Administration-approved contraceptive method in accordance with the product label)
6. Participant must have been either previously enrolled in the phase 3 mRNA-1273 COVE trial^23,24^, must have received two doses of mRNA-1273 in that study, with his/her second dose at least 6 months before enrollment in this study, and must be currently enrolled and compliant in that study (that is, has not withdrawn or discontinued early); or participant must have received two doses of mRNA-1273 under the EUA with their second dose at least 6 months before enrollment in this study; or have received a two-dose primary series of mRNA-1273 followed by a 50 µg booster dose of mRNA-1273 in the mRNA-1273 COVE trial or under EUA at least 3 months before enrollment this study; and able to provide proof of vaccination status at the time of screening (day 1).

#### Exclusion criteria

Participants meeting any of the following criteria at the Screening Visit, unless noted otherwise, will be excluded from the study:

1. Had substantial exposure to someone with SARS-CoV-2 infection or coronavirus disease 2019 (COVID-19) in the past 14 days, as defined by the CDC as a close contact of someone who has COVID-19).
2. Has known history of SARS-CoV-2 infection within 3 months before enrollment.
3. Is acutely ill or febrile (temperature ≥38.0 °C (100.4 °F)) less than 72 hours before or at the Screening Visit or day 1. Participants meeting this criterion may be rescheduled and will retain their initially assigned participant number.
4. Currently has symptomatic acute or unstable chronic disease requiring medical or surgical care, to include substantial change in therapy or hospitalization for worsening disease, at the discretion of the investigator.
5. Has a medical, psychiatric or occupational condition that may pose additional risk as a result of participation, or that could interfere with safety assessments or interpretation of results according to the investigator’s judgment.
6. Has a current or previous diagnosis of immunocompromising condition to include human immunodeficiency virus, immune-mediated disease requiring immunosuppressive treatment or other immunosuppressive condition.
7. Has received systemic immunosuppressants or immune-modifying drugs for >14 days in total within 6 months before Screening (for corticosteroids ≥10 mg day^−1^ of prednisone equivalent) or is anticipating the need for immunosuppressive treatment at any time during participation in the study.
8. Has known or suspected allergy or history of anaphylaxis, urticaria or other important adverse reaction to the vaccine or its excipients.
9. Has a documented history of myocarditis or pericarditis within 2 months before Screening Visit (day 0).
10. Coagulopathy or bleeding disorder considered a contraindication to intramuscular (IM) injection or phlebotomy.
11. Has received or plans to receive any licensed vaccine ≤28 days before the injection (day 1) or a licensed vaccine within 28 days before or after the study injection, with the exception of influenza vaccines, which may be given 14 days before or after receipt of a study vaccine.
12. Has received systemic immunoglobulins or blood products within 3 months before the Screening Visit (day 0) or plans for receipt during the study.
13. Has donated ≥450 ml of blood products within 28 days before the Screening Visit or plans to donate blood products during the study.
14. Plans to participate in an interventional clinical trial of an investigational vaccine or drug while participating in this study.
15. Is an immediate family member or household member of study personnel, study site staff or sponsor personnel.
16. Is currently experiencing an SAE in COVE trial at the time of screening for this study.

### Trial vaccine

The bivalent mRNA-1273.222 50 µg vaccine contains equal amounts of two mRNAs (25 µg of each mRNA sequence) that encode the prefusion-stabilized spike glycoproteins of the ancestral SARS-CoV-2 (Wuhan-Hu-1) and the Omicron variants (BA.4 and BA.5 have identical sequences and are designated as BA.4/BA.5). The monovalent mRNA-1273 50 µg original (Moderna COVID-19) vaccine)^23,24^ contains a single mRNA sequence encoding the spike glycoprotein of ancestral SARS-CoV-2 (Wuhan-Hu-1). In both vaccines, mRNAs are encapsulated in lipid nanoparticles as described previously^35^. The booster doses of mRNA-1273.222 and mRNA-1273 were administered by IM injection at doses of 50 µg of mRNA in a 0.5 ml volume.

### Safety assessment

The primary safety objective was to evaluate the safety and reactogenicity of 50 µg mRNA‑1273.222 when administered as a second booster dose. Safety assessments included solicited local and systemic adverse reactions ≤7 days and unsolicited adverse events ≤28 days post booster administration, and serious adverse events, adverse events leading to discontinuation from study vaccine and/or participation, medically attended adverse events and adverse events of special interest from day 1 through the entire study period (~6 months).

### Immunogenicity assessment

#### Immunogenicity objectives and endpoints

The prespecified primary immunogenicity objectives (Supplementary Table 1 and Supplementary Fig. 1) were to demonstrate noninferior nAb or superior nAb responses against Omicron BA.4/BA.5 and noninferior nAb responses against ancestral SARS-CoV-2 with the D614G mutation (ancestral SARS-CoV-2 (D614G)), 28 days after the second booster dose (day 29) of mRNA-1273.222 50 µg compared with mRNA-1273 50-µg (Statistical Analysis). Noninferiority objectives were based on endpoints of GMR and SRR differences and those for superiority were based on GMR.

nAb GMTs at inhibitory dilutions 50% (ID_50_) were assessed using validated SARS-CoV-2 spike-pseudotyped lentivirus neutralization assays against pseudoviruses containing the SARS-CoV-2 full-length spike proteins of ancestral SARS-CoV-2 (D614G), or Omicron variants BA.4/BA.5, BQ.1.1 and XBB.1 variants. GM levels of spike-binding antibody (bAb) were also assessed using an (Meso Scale Discovery (MSD)) assay against ancestral SARS-CoV-2 (D614G), Gamma (P.1), Alpha (B.1.1.7), Delta [B.1.617.2; AY.4] and Omicron (BA.1) variants.

#### Immunogenicity assays

##### SARS-CoV-2 spike-pseudotyped virus neutralization assay

SARS-CoV-2 nAb in samples were assessed using a validated SARS-CoV-2 spike (S)-pseudotyped virus neutralization assay (PsVNA) in 293/ACE2 cells^14^. The PsVNA quantifies nAb using lentivirus particles that express full-length spike proteins on their surface, and contain a firefly luciferase reporter gene for quantitative measurements of infection in transduced 293T cells expressing high levels of ACE2 (293T/ACE2 cells) by relative luminescence units (RLU). Serial dilution of antibodies was used to produce a dose−response curve. Neutralization was measured as the serum dilution at which RLU was reduced by 50% (ID_50_) relative to mean RLU in virus control wells (cells + virus but no sample) after subtraction of mean RLU in cell control wells (cells only). Positive controls were included on each assay plate to follow stability over time.

The spike-pseudotyped viruses were derived from ancestral Wuhan-Hu-1 with the following amino acid substitutions: (prototype (D614G); Omicron subvariants BA.4 and BA.5 (designated BA.4/BA.5 for identical spike sequences between BA.4 and BA.5 (T191, L24S, ΔP25, ΔP26, ΔA27, G142D, V213G, G339D, S371F, S373P, S375F, T376A, D405N, R408S, K417N, N440K, L452R, S477N, T478K, E484A, F486V, Q498R, N501Y, Y505H, D614G, H655Y, N679K, P681G, N764K, D796Y, Q954H, N969K])); Omicron subvariant BQ.1.1 (T19I, L24S, ΔP25, ΔP26, ΔA27, H69-, V70-, G142D, V213G, G339D, R346T, S371F, S373P, S375F, T376A, D405N, R408S, K417N, N440K, K444T, L452R, N460K, S477N, T478K, E484A, F486V, Q498R, N501Y, Y505H, D614G, H655Y, N679K, P681H, N764K, D796Y, Q954H, N969K); Omicron subvariant XBB.1 (T19I, L24S, ΔP25, ΔP26, ΔA27, V83A, G142D, Y145Q, ΔH146, Q183E, V213E, G252V, G339H, R346T, L368I, S371F, S373P, S375F, T376A, D405N, R408S, K417N, N440K, V445P, G446S, N460K, S477N, T478K, E484A, F486S, F490S, Q498R, N501Y, Y505H, D614G, H655Y, N679K, P681H, N764K, D796Y, Q954H, N969K)).

##### SARS-CoV-2 MSD assay

The validated MSD assay (SARSCOV2S2P (VAC123); https://www.mesoscale.com/products/v-plex-sars-cov-2-panel-24-igg-kit-k15575u/) uses an indirect, quantitative, electrochemiluminescence method to detect SARS-CoV-2 binding IgG antibodies that bind to the SARS-CoV-2 full-length spike protein (Wuhan-Hu-1 ancestral SARS-CoV-2; beta (B.1.351) with the following amino acid changes in the spike protein (L18F, D80A, D215G, Δ242-244, R246I, K417N, E484K, N501Y, D614G and A701V); Alpha (B.1.1.7) with the following amino acid changes in the spike protein (ΔH69-V70, ΔY144, N501Y, A570D, D614G, P681H, T761I, S982A and D1118H); Gamma (P.1) with the following amino acid changes in the spike protein (L18F, T20N, P26S, D138Y, R190S, K417T, E484K, N501Y, D614G, H655Y, T1027I and V1176F)); Delta (B.1.617.2; AY.4; Alt Seq 2) with the following amino acid changes in the spike protein (T19R, T95I, G142D, Δ156/157, R158G, L452R, T478K, D614G, P681R and D950N)); Omicron (B.1.1.529; BA.1) with the following amino acid changes in the spike protein (A67V, ∆H69-V70, T95I, G142D, ∆143-145, ∆211/L212I, ins214EPE, G339D, S371L, S373P, S375F, K417N, N440K, G446S, S477N, T478K, E484A, Q493R, G496S, Q498R, N501Y, Y505H, T547K, D614G, H655Y, N679K, P681H, N764K, D796Y, N856K, Q954H, N969K and L981F)) in human serum. The assay was performed by PPD (Thermo Fisher Scientific Vaccines Laboratory Services). The assay is based on MSD technology which employs capture molecule MULTI-SPOT microtiter plates fitted with a series of electrodes.

### Incidence of SARS-CoV-2 infections

The incidence of symptomatic and asymptomatic SARS-CoV-2 infection was an exploratory objective (Supplementary Table 1). Vaccine effectiveness was not assessed in this trial, but COVID-19 and SARS-CoV-2 infection were actively surveilled through weekly contact and blood draws. SARS-CoV-2 infection is a combination of symptomatic infection (COVID-19) and asymptomatic SARS-CoV-2 infection for participants with negative SARS-CoV-2 status prebooster. Symptomatic infection was evaluated using the primary case definition in the COVE study^24,36^ and a secondary case definition based on the Centers for Disease Control and Prevention (CDC) criteria^37^. Asymptomatic SARS-CoV-2 infection was defined as a positive RT–PCR test or a positive serologic test for antinucleocapsid antibody after a negative test at the time of enrollment, in the absence of symptoms.

### Statistical analysis

Statistical analysis populations are detailed in Supplementary Table 2 and Fig. 1b. Safety was evaluated in the safety set consisting of all participants who received mRNA-1273.222 and solicited adverse reactions in all participants and by prebooster SARS-CoV-2 infection status in the solicited safety set using the same methodology as described for the safety evaluation of the 50 µg mRNA-1273 comparator group^5,6^. The per-protocol immunogenicity set consists of all participants who received the planned booster doses, had prebooster and day 29 antibody data available and no major protocol deviations. Primary immunogenicity objectives were assessed in the per-protocol immunogenicity-SARS-CoV-2-negative set (primary analysis set), those participants without evidence of prior SARS-CoV-2 infection (negative RT–PCR and negative binding antibody against SARS-CoV-2 nucleocapsid at prebooster day 1). Analyses were also performed in participants who had evidence of SARS-CoV-2 infection prebooster (positive binding antibody against SARS-CoV-2 nucleocapsid or positive RT–PCR at booster day 1).

#### Immunogenicity analysis

The primary immunogenicity objectives were evaluated using a prespecified hierarchical approach which compared 50 µg mRNA-1273.222 to 50 µg mRNA-1273 (active control arm in part F, Cohort 2) as second booster doses (Supplementary Fig. 1). For the primary objective on immune response, five hypotheses were specified to be evaluated at day 29 post booster (online protocol and SAP and Supplementary Fig. 1). Interim analysis results for the day 29 hypotheses are presented in this report. Below are the five hypotheses for day 29:

1. 50 µg mRNA-1273.222, as a second booster dose, against Omicron BA.4/5 is noninferior to the second booster dose of (50 µg) mRNA-1273 against Omicron BA.4/5 based on the GMT ratio of mRNA-1273.222 against Omicron BA.4/5 at Day 29 compared to mRNA-1273 against Omicron BA.4/5 at Day 29 with a noninferiority margin of 1.5.
2. 50 μg mRNA-1273.222, as a second booster dose, against Omicron BA.4/5 is noninferior to the second booster dose of (50 μg) mRNA-1273 against Omicron BA.4/5 based on the difference in SRR at Day 29 with a noninferiority margin, if
  the lower bound of the 95% CI of the SRR difference (50 μg mRNA-1273.222 against Omicron BA.4/5 at Day 29 - 50 μg mRNA-1273 against Omicron BA.4/5 at Day 29) is >-5%, then the noninferiority of mRNA-1273.222 against Omicron BA. 4/5 compared to that of mRNA-1273 is demonstrated based on a noninferiority margin of 5%.
  the lower bound of the 95% CI of the seroresponse difference (50 μg mRNA-1273.222 against Omicron BA.4/5 at day 29 - 50 μg mRNA-1273 against Omicron BA.4/5 at day 29) is >-10% but  ≤-5%, then noninferiority of mRNA-1273.222 against Omicron BA. 4/5 compared to that of mRNA-1273 is demonstrated based on a noninferiority margin of 10%.
3. 50 μg mRNA-1273.222, as a second booster dose, against the ancestral SARS-CoV-2 D614G is noninferior to the second booster dose of (50 μg) mRNA-1273 against the ancestral SARS-CoV-2 D614G based on the GMT ratio of mRNA-1273.222 against the ancestral SARS-CoV-2 D614G at day 29 compared to mRNA-1273 against the ancestral SARS-CoV-2 D614G at day 29 with a noninferiority margin of 1.5.
4. 50 μg mRNA-1273.222, as a second booster dose, against the ancestral SARS-CoV-2 D614G is noninferior to the second booster dose of (50 μg) mRNA-1273 against ancestral SARS-CoV-2 D614G based on the difference in SRR at day 29 with a noninferiority margin of 10%.
5. 50 μg mRNA-1273.222, as a second booster dose, against the Omicron BA.4/BA.5 is superior to the second booster dose of (50 μg) mRNA-1273 against Omicron BA.4/BA.5 day 29.

The primary immunogenicity objectives were assessed in the per-protocol immunogenicity-SARS-CoV-2-negative set with a target enrollment of approximately 500 participants for 50 μg mRNA-1273.222. Assuming 40% of participants were excluded from the per-protocol immunogenicity-SARS-CoV-2-negative set (due to a SARS-CoV-2 infection prebooster), with approximately 300 participants in the 50 μg mRNA-1273.222 and 260 participants in the 50 μg mRNA-1273 (Part F, Cohort 2–50 μg mRNA-1273) arms in the per-protocol immunogenicity-SARS-CoV-2-negative set, there is approximately 60% power to demonstrate the primary immunogenicity objectives with an alpha of 0.05 (two-sided) at day 29. The assumptions were that the true GMR (mRNA-1273.222 second booster versus mRNA-1273 second booster) against Omicron BA.4/5 is 1.5, GMR (mRNA-1273.222 second booster versus mRNA-1273 second booster) against ancestral SARS-CoV-2 D614G is 1, the standard deviation of the log-transformed titer is 1.5 and the noninferiority margin for GMR is 1.5. The true SRR against Omicron BA.4/5 after mRNA-1273.222 as a second booster dose is 95% (same assumption for 50 μg mRNA-1273), and noninferiority margin for SRR difference against Omicron BA.4/5 is 5%. The true SRR against ancestral SARS-CoV-2 D614G after mRNA-1273.222 as a second booster dose is 95% (same assumption for 50 μg mRNA-1273), and the noninferiority margin for SRR difference against ancestral SARS-CoV-2 D614G is 10%.

For the primary immunogenicity objective, an interim analysis was planned at day 29 with a two-sided alpha (0.05) allocated for immunogenicity hypothesis testing. The primary immunogenicity objective was considered met for noninferiority when the lower bound of the 95 CI of the GMR was >0.667 and the SRR difference is >-10%. Superiority was considered met when the lower bound of the 95 CI of GMR is >1. These noninferiority and superiority criteria were chosen based on FDA guidelines^19,38^. Day 29 interim analysis results are presented. The within-study noncontemporaneous mRNA-1273 comparator group (Part F, cohort 2) was enrolled during 18 February–8 March 2022 and data for this group, based on the data cutoff date of 6 July 2022 at the day 91 interim analysis, is used for the immunogenicity comparison. The superiority of the antibody response against Omicron BA.4/BA.5 after a second booster dose of 50 µg mRNA‑1273.222 compared with 50 µg mRNA‑1273 was evaluated only after meeting noninferiority criteria for the four primary objectives^39^: noninferiority of the antibody response against BA.4/BA.5 after the second booster doses of 50 µg mRNA‑1273.222 versus 50 µg mRNA‑1273 based on GMR (1) and SRR difference (2), and noninferiority of the antibody response against ancestral SARS-CoV-2 (D614G) after the second booster doses of 50 µg mRNA‑1273.222 versus 50 µg mRNA‑1273 based on GMR (3) and SRR difference (4).

Observed GMTs (95% CI) using *t*-distribution of log-transformed antibody titers are presented. Differences in antibody responses between the mRNA-1273.222 and mRNA-1273 groups were assessed using an analysis of covariance (ANCOVA) model (antibody titers post booster as dependent variable, group variable mRNA-1273.222 and mRNA-1273 as the fixed effect) adjusted for age groups (<65, ≥65 years) and prebooster antibody titers. The GMTs (95% CI) estimated by the geometric least squares mean from the model and differences in antibody responses (GMR) between groups estimated by the ratio of geometric least mean square (95% CIs) are provided. The 95% CI for GMR was used to assess the between-group difference in antibody responses.

Seroresponse is defined as ≥4 × lower limit of quantification (LLOQ) for those with baseline <LLOQ; ≥fourfold rise for those baseline ≥LLOQ. Seroresponse was derived based on prevaccination (preinjection 1 of the primary series) and prebooster baselines Seroresponse, based on change (fold-rise) from preinjection 1 of the primary series, was considered the primary approach for seroresponse. For participants without preinjection 1 antibody titer information, seroresponse was defined as ≥4× LLOQ for those with negative SARS-CoV-2 status at preinjection 1 of the primary series, and antibody titers were imputed as <LLOQ at preinjection 1 of primary series. For participants who were without SARS-CoV-2 status information at preinjection 1 of the primary series, their prebooster SARS-CoV-2 status was used to impute their SARS-CoV-2 status at preinjection 1.

The SRR of each arm against ancestral SARS-CoV-2 (D614G) and variants, defined as the percentage of participants achieving seroresponse against ancestral SARS-CoV-2 (D614G) and variants, respectively, are provided for each arm with the 95% CI calculated using the Clopper–Pearson method. The differences of SRR between mRNA-1273.222 and mRNA-1273 were calculated with 95% CI based on the stratified Miettinen–Nurminen method adjusting for age groups.

An analysis of the primary immunogenicity endpoints was also performed in the per-protocol set for immunogenicity (participants with and without evidence of prior SARS-CoV-2 infection prebooster), using an ANCOVA model, with antibody titers at day 29 post booster as the dependent variable and the vaccine group variable as the fixed effect, adjusting for age groups (<65, ≥65 years), prebooster SARS-CoV-2 infection status and prebooster titers. The seroresponse difference between the mRNA-1273.222 and mRNA-1273 groups was calculated with 95% CI based on stratified Miettinen–Nurminen method adjusted for the prebooster SARS-CoV-2 infection status and age group. A preplanned subgroup analysis of participants with prior evidence of SARS-CoV-2 infection prebooster was performed using an ANCOVA model to assess nAb differences between the mRNA-1273.222 and mRNA-1273 groups based on GMRs with 95% CIs. Lastly, a sensitivity analysis was performed excluding the participants with evidence of SARS-CoV-2 infection after the booster dose. Evidence of prior SARS-CoV-2 infection was defined as a negative binding antibody test against SARS-CoV-2 nucleocapsid and a negative RT–PCR at booster day 1 and participants who had evidence of SARS-CoV-2 infection defined as a positive binding antibody test against SARS-CoV-2 nucleocapsid or positive RT–PCR at booster day 1.

Immunogenicity analyses included assessment of the observed bAb GM levels against SARS-CoV-2 variants and bAb comparisons between mRNA-1273.222 and mRNA-1273 based on ANCOVA-estimated antibody levels and GMRs (95% CIs). A posthoc analysis of immunogenicity against Omicron BQ.1.1 and XBB.1 variants that emerged during the study was additionally explored in random samples of recipients (*n* = 60) stratified by age and baseline prebooster titers from the full analysis sets of the mRNA-1273.222 group (Supplementary Table 3 and random sampling method table below). Among those with negative SARS-CoV-2 prebooster status, participants were first stratified by age (every 5 years from 20–90 years) and by deciles of baseline prebooster BA.4/BA.5 titers, then randomly selected as 40 pairs (*n* = 40/each group; *n* = 20 each from age groups <65 and ≥65 years) from two booster groups. Participants who were SARS-CoV-2 positive were stratified by age (every 5 years from 20–90 years) and by quartiles of baseline prebooster BA.4/BA.5 titers, then randomly selected in 20 pairs in each booster group (*n* = 20; *n* = 10 each from age groups <65 and ≥65 years).

**Table:** 

Sampling method	SARS-CoV-2 negative *n* = 40			SARS-CoV-2 positive *n* = 20		
Baseline covariate strata	Age (every 5 years from 20–90 years) and baseline prebooster BA.4/BA.5 titer deciles			Age (every 5 years from 20–90 years) and baseline prebooster BA.4/BA.5 titer quartiles		
	18–64	≥65	All	18–64	≥65	All
**mRNA-1273.222 (*n*)**	20	20	40	10	10	20

Pseudovirus neutralizing antibodies against BQ.1.1, XBB.1 and XBB.1.5 were assessed in subsets of recipients (*n* = 60) stratified by age and baseline prebooster titers from the mRNA-1273.222 group in the full analysis set. Among those with negative SARS-CoV-2 prebooster status, participants were first stratified by age (every 5 years from 20–90 years) and by deciles of baseline prebooster BA.4/BA.5 titers and participants who were SARS-CoV-2 positive were stratified by age (every 5 years from 20–90 years) quartiles of baseline prebooster BA.4/BA.5 titers. Pairs of participants were then randomly selected from age groups <65 and ≥65 years for the analysis.

Additionally, the effect of time intervals between the first booster dose of mRNA-1273 and second booster doses of mRNA-1273 and mRNA-1273.222 on neutralizing antibody responses against Omicron BA.4/BA.5 and ancestral SARS-CoV-2 (D614G) post boost were explored in participants without previous infection grouped by quartiles of dosing intervals within each booster vaccine group, and GMT and GMFR at day 29 were summarized.

#### Analysis of incidence of SARS-CoV-2 infections

The primary analysis population to assess the incidence of symptomatic SARS-CoV-2 infection (COVID-19), asymptomatic SARS-CoV-2 infection and SARS-CoV-2 infection was the primary set for efficacy. The number and percentage of participants with asymptomatic or symptomatic SARS-CoV-2 infection and COVID-19-events post booster are summarized starting 14 days post booster regardless of prebooster SARS-CoV-2 infection status.

##### SARS-CoV-2 infection

SARS-CoV-2 infection was defined in participants with negative SARS-CoV-2 status prebooster by either bAb levels against SARS-CoV-2 nucleocapsid protein (as measured by Roche Elecsys^20^) at day 1 that became positive starting at day 29 or later, OR a positive RT–PCR starting 14 days after the booster dose.

For the analysis, documented infection was counted starting 14 days after the booster dose, which required a positive serology test result based on bAb specific to SARS-CoV-2 nucleocapsid at day 29 or later, or a positive RT–PCR result starting 14 days after the booster dose. The date of documented infection was the earlier of the date of a positive post baseline RT–PCR result or a positive serology test result based on bAb specific to SARS-CoV-2 nucleocapsid. The time to first SARS-CoV-2 infection was calculated as the date of the first documented infection minus the date of injection +1. Cases were counted starting 14 days after the injection (date of documented infection minus date of the injection ≥14). SARS-CoV-2 infection cases are summarized based on tests performed at least 14 days after the booster dose.

##### Asymptomatic SARS-CoV-2 infection

Asymptomatic SARS-CoV-2 infection was measured by RT–PCR of nasal swabs and/or serology tests obtained at post baseline study visits counted starting 14 days after the injection in participants with negative SARS-CoV-2 status prebooster. Asymptomatic SARS-CoV-2 infection was identified by the absence of symptoms and infections as detected by RT–PCR or serology tests. Specifically, the absence of COVID-19 symptoms AND at least either a positive serology test result based on bAb specific to SARS-CoV-2 nucleocapsid protein day 29 or later, when blood samples for immunogenicity are collected, or a positive RT–PCR test at scheduled or unscheduled/illness visits. The date of documented asymptomatic infection was the earlier date of positive serology test result based on bAb specific to SARS-CoV-2 nucleocapsid due to infection, or positive RT–PCR, with absence of symptoms. The time to the asymptomatic SARS-CoV-2 infection was calculated as the date of asymptomatic SARS-CoV-2 infection minus the date of injection +1.

##### Symptomatic SARS-CoV-2 infection (COVID-19)

Symptomatic SARS-CoV-2 infection (COVID-19) was defined as the incidence of the first occurrence of symptomatic SARS-CoV-2 infection measured by RT–PCR of nasal swabs starting 14 days after the booster dose. Surveillance for COVID-19 symptoms is conducted via weekly contact and blood draw, and an illness visit to collect a nasopharyngeal swab was arranged for participants reporting COVID-19 symptoms.

Two definitions of symptomatic SARS-CoV-2 infection (COVID-19) were used including the primary case definition in the COVE trial^23,24^ based on a positive post baseline RT–PCR result AND at least TWO systemic symptoms (fever (≥38 °C/≥100.4 °F), chills, muscle and/or body aches (not related to exercise), headache, sore throat, new loss of taste/smell; OR at least ONE of respiratory signs/symptoms (cough, shortness of breath and/or difficulty breathing, OR clinical or radiographical evidence of pneumonia). The second case definition is based on CDC criteria for symptomatic disease defined as a positive post baseline RT–PCR test AND at least ONE systemic or respiratory symptoms (fever (≥38 °C/≥100.4 °F), chills, cough, shortness of breath and/or difficulty breathing, fatigue, muscle and/or body aches (not related to exercise), headache, new loss of taste/smell, sore throat, congestion, runny nose, nausea, vomiting or diarrhea)^37^.

The date of a documented COVID-19 case was the later date of a symptom and the date of a positive RT–PCR test, and the two dates were to be within 14 days of each other. The time to the first occurrence of COVID-19 is calculated as the date of documented COVID-19 minus the date of injection +1. Cases are counted starting 14 days after the injection (date of documented COVID-19 minus date of the injection ≥14).

All analyses were conducted using SAS v.9.4 or higher.

### Reporting summary

Further information on research design is available in the Nature Portfolio Reporting Summary linked to this article.

## Online content

Any methods, additional references, Nature Portfolio reporting summaries, source data, extended data, supplementary information, acknowledgements, peer review information; details of author contributions and competing interests; and statements of data and code availability are available at 10.1038/s41591-023-02517-y.

## Supplementary information

Supplementary Information List of study investigators, Supplementary Figs. 1 and 2, Tables 1–12, References, Study protocol and SAP.

Reporting Summary

## Source data

Source Data Fig. 1 Source data for Figs. 1 and 2 and Extended Data Figs. 2 and 3.

## Data Availability

Data associated with this study are provided in the paper or supplementary materials. Source data for Figs. 2 and 3 and Extended Data Figs. 2 and 3 and the protocol and statistical analysis plan are provided as Supplementary Information. SARS-COV-2 variant sequences were obtained from GISAID Overview of Variants/Mutations, https://covariants.org/variants (2023). As the trial is ongoing, access to patient-level data and supporting clinical documents by qualified external researchers may be available upon request and subject to review once the trial is complete. A materials transfer and/or data access agreement with the sponsor will be required for accessing shared data. Such requests can be made to Dr. Spyros Chalkias, Moderna Inc., 200 Technology Square, Cambridge, MA 02139, USA. Source data are provided with this paper.
