# Supplementary material for: Original SARS-CoV-2 monovalent and Omicron BA.4/BA.5 bivalent COVID-19 mRNA vaccines: phase 2/3 trial interim results
Source: Nat Med. 2023 Aug 31;29(9):2325–33. doi: 10.1038/s41591-023-02517-y (PMC10504066; doi:10.1038/s41591-023-02517-y)
Supplement: Supplementary file 2 — Reporting Summary [file 41591_2023_2517_MOESM2_ESM.pdf]

nature portfolio

Corresponding author(s): Spros Chalkias

Last updated by author(s): 08-07-23

Reporting Summary

Nature Portfolio wishes to improve the reproducibility of the work that we publish. This form provides structure for consistency and transparency in reporting. For further information on Nature Portfolio policies, see our Editorial Policies and the Editorial Policy Checklist.

Statistics

For all statistical analyses, confirm that the following items are present in the figure legend, table legend, main text, or Methods section.

n/a	Confirmed	
☐	☒	The exact sample size (*n*) for each experimental group/condition, given as a discrete number and unit of measurement
☐	☒	A statement on whether measurements were taken from distinct samples or whether the same sample was measured repeatedly
☐	☒	The statistical test(s) used AND whether they are one- or two-sided<br>*Only common tests should be described solely by name; describe more complex techniques in the Methods section.*
☒	☐	A description of all covariates tested
☒	☐	A description of any assumptions or corrections, such as tests of normality and adjustment for multiple comparisons
☐	☒	A full description of the statistical parameters including central tendency (e.g. means) or other basic estimates (e.g. regression coefficient) AND variation (e.g. standard deviation) or associated estimates of uncertainty (e.g. confidence intervals)
☐	☒	For null hypothesis testing, the test statistic (e.g. *F*, *t*, *r*) with confidence intervals, effect sizes, degrees of freedom and *P* value noted<br>*Give P values as exact values whenever suitable.*
☒	☐	For Bayesian analysis, information on the choice of priors and Markov chain Monte Carlo settings
☐	☒	For hierarchical and complex designs, identification of the appropriate level for tests and full reporting of outcomes
☐	☒	Estimates of effect sizes (e.g. Cohen's *d*, Pearson's *r*), indicating how they were calculated

Our web collection on statistics for biologists contains articles on many of the points above.

Software and code

Policy information about availability of computer code

Data collection	Not applicable. Software was not used in data collection.
Data analysis	All analyses were conducted using SAS Version 9.4 or higher.

For manuscripts utilizing custom algorithms or software that are central to the research but not yet described in published literature, software must be made available to editors and reviewers. We strongly encourage code deposition in a community repository (e.g. GitHub). See the Nature Portfolio guidelines for submitting code & software for further information.

Data

Policy information about availability of data

All manuscripts must include a data availability statement. This statement should provide the following information, where applicable:

- Accession codes, unique identifiers, or web links for publicly available datasets
- A description of any restrictions on data availability
- For clinical datasets or third party data, please ensure that the statement adheres to our policy

Data associated with this study are provided in the paper or supplementary materials. Source data for Figures 2-3 and Extended data Figures 2-3 and the protocol and statistical analysis plan are provided as supplementary materials. SARS-COV-2 variant sequences were obtained from GISAID Overview of Variants/Mutations, https://covariants.org/variants (2023). As the trial is ongoing, access to patient-level data and supporting clinical documents by qualified external researchers may

be available upon request and subject to review once the trial is complete. Such requests can be made to Dr. Spyros Chalkias, Moderna Inc., 200 Technology Square, Cambridge, MA 02139, USA.

Human research participants

Policy information about studies involving human research participants and Sex and Gender in Research.

Reporting on sex and gender	There were 62% and 51% female participants in the 50-µg mRNA-1273.222 and 50-µg mRNA-1273 groups, respectively. Gender was self-reported at enrollment.
Population characteristics	Participant demographics and baseline characteristics were generally balanced. Mean ages were 50.8 and 57.6 years, and 62% and 51% were female in the 50-µg mRNA-1273.222 and 50-µg mRNA-1273 groups, respectively. Most participants were White (83% and 86%),11% and 7% were Black/African American and 11% and 10% were of Hispanic/Latinx ethnicity in the mRNA-1273.222 and mRNA-1273 groups, respectively. The percentages of participants with SARS-CoV-2-infection pre-booster were 56% in the mRNA-1273.222 and 27% in the mRNA-1273 groups.
Recruitment	Participants were recruited to the sites through review of unit past participant logs for eligible individuals based on the inclusion and exclusion criteria, public advertisements using social media, and referrals from primary care physicians. As such, there are not believed to beany self-selection biases that would impact results. Participants were enrolled in a sequential, non-randomized manner and received single-second boosters of 50-µg mRNA-1273 (enrolled between February 18th and March 8th, 2022) or 50-µg bivalent mRNA-1273.222 (enrolled between August 10th and 23rd, 2022). These participants had previously received the primary series of 100-µg mRNA-1273 and a first booster dose of 50-µg mRNA-1273, ≥3 months before enrollment. Of these, 305 (59.7%) participants originated from the COVE trial and 206 (40.3%) were US vaccinees under the EUA, respectively. Given that enrollment with the original vaccine mRNA-1273 was no longer feasible after the US FDA recommendation and authorization of bivalent booster vaccine, a mRNA-1273 comparator group was not enrolled. Instead, a within-study, non-contemporaneous mRNA-1273 group (n=379; enrollment February 18th-March 8th, 2022 data cut-off date of July 6, 2022 at the day 91 interim analysis) was used for the immunogenicity comparison.
Ethics oversight	The trial is being conducted in accordance with the International Council for Harmonisation of Technical Requirements for Registration of Pharmaceuticals for Human Use, Good Clinical Practice guidelines. The central Institutional Review Board/ Ethics Committee (Advarra, Inc., 6100 Merriweather Drive, Columbia, MD 21044) approved the protocol and consent forms.

Note that full information on the approval of the study protocol must also be provided in the manuscript.

Field-specific reporting

Please select the one below that is the best fit for your research. If you are not sure, read the appropriate sections before making your selection.

☒ Life sciences ☐ Behavioural & social sciences ☐ Ecological, evolutionary & environmental sciences

For a reference copy of the document with all sections, see nature.com/documents/nr-reporting-summary-flat.pdf

Life sciences study design

All studies must disclose on these points even when the disclosure is negative.

Sample size	The primary immunogenicity objectives were assessed in the per-protocol immunogenicity–SARS-CoV-2-negative set (PPSI-Neg), consisting of SARS-CoV-2 negative participants who have no serologic or virologic evidence of SARS-CoV-2 infection at baseline pre-booster, defined as having both a negative RT-PCR test for SARS-CoV-2 and a negative serology test based on binding antibody specific to SARS-CoV-2 nucleocapsid protein. The target enrollment was approximately 500 participants for 50 µg mRNA 1273.222. Assuming 40% of participants are excluded from the PPSI-Neg (due to a SARS-CoV-2 infection pre-booster), with approximately 300 participants in 50 µg mRNA-1273.222 and 260 participants in 50 µg mRNA-1273 (Part F, Cohort 2-50 µg mRNA-1273) in the PP Set for Immunogenicity and SARS-CoV-2 negative, there is approximately 60% power to demonstrate the primary immunogenicity objectives with an alpha of 0.05 (2-sided) at day 29. The assumptions are: the true GMR (mRNA-1273.222 second booster vs. mRNA-1273 second booster) against omicron BA.4/5 is 1.5, GMR (mRNA-1273.222 second booster vs. mRNA-1273 second booster) against ancestral SARS-CoV-2 D614G is 1, the standard deviation of the log-transformed titer is 1.5, and the non-inferiority margin for GMR is 1.5. The true SRR against omicron BA.4/5 after mRNA-1273.222 as a second booster dose is 95% (same assumption for 50 µg mRNA-1273), and non-inferiority margin for SRR difference against omicron BA.4/5 is 5%. The true SRR against ancestral SARS-CoV-2 D614G after mRNA-1273.222 as a second booster dose is 95% (same assumption for 50 µg mRNA-1273), and the non-inferiority margin for SRR difference against ancestral SARS-CoV-2 D614G is 10%.
Data exclusions	In the 50-µg mRNA-1273 arm, 379 participants were enrolled and received mRNA-1273; two participants had previously received the primary series but not a first booster dose and another participant had a major protocol deviation, and three were excluded from all analysis sets. Seven participants in the mRNA-1273.222 arm, and 8 participants in the mRNA-1273 arm in the per-protocol immunogenicity set had missing pre-booster SARS-CoV-2 information and were excluded from the PPSI-SARS-CoV-2-negative and PPSI-SARS-CoV-2-positive groups.
Replication	In this clinical study, one sample at each time point was collected for trial participants.
Randomization	This is an open-label study and participants were not randomized to study vaccine.

Blinding

This is an open-label trial and blinding is not relevant.

Reporting for specific materials, systems and methods

We require information from authors about some types of materials, experimental systems and methods used in many studies. Here, indicate whether each material, system or method listed is relevant to your study. If you are not sure if a list item applies to your research, read the appropriate section before selecting a response.

Materials & experimental systems

n/a	Involved in the study
☒	☐ Antibodies
☐	☒ Eukaryotic cell lines
☒	☐ Palaeontology and archaeology
☒	☐ Animals and other organisms
☐	☒ Clinical data
☒	☐ Dual use research of concern

Methods

n/a	Involved in the study
☒	☐ ChIP-seq
☒	☐ Flow cytometry
☒	☐ MRI-based neuroimaging

Eukaryotic cell lines

Policy information about cell lines and Sex and Gender in Research

Cell line source(s)

Neutralization assays were performed using stably transduced 293T cells (human embryonic kidney cells in origin; American Type Culture Collection, cat. no. CRL-11268) expressing high levels of ACE2 (293T/ACE2 cells) obtained from Drs. Mike Farzan and Huihui Mu at Scripps as previously described (Gilbert PB, et al. Science 2022;375:43-50).

Authentication

The cell lines were not authenticated.

Mycoplasma contamination

The cell lines tested negative for mycoplasma contamination.

Commonly misidentified lines
(See ICLAC register)

No misidentifed cell lines were used in the study.

Clinical data

Policy information about clinical studies

All manuscripts should comply with the ICMJE guidelines for publication of clinical research and a completed CONSORT checklist must be included with all submissions.

Clinical trial registration

Clintrials.govNCT04927065

Study protocol

The protocol and SAP are provided in supplementary material.

Data collection

The trial is ongoing and being conducted across 23 medical sites in the US (provided in the supplement). Participants were enrolled during August 10th-23rd, 2022 for mRNA-1273.222 (Part H) and during February 18th-March 8th, 2022 for mRNA-1273 (Part F, cohort 2). Between August 10 and 23 2022, 511 participants received 50-µg of mRNA-1273.222. These participants had previously received the primary series of 100-µg mRNA-1273 and a first booster dose of 50-µg mRNA-1273, ≥3 months before enrollment (Figure 1). Of these,305 (59.7%) participants originated from the COVE trial and 206 (40.3%) were US vaccinees under the EUA, respectively. Data is reported for the mRNA-1273.222 (Part H) group based on the 29-day interim analysis data cutoff date of September 23, 2022 and in the mRNA-1273group (Part F, cohort 2) based on the data cutoff date of July 6, 2022 at the day 91 interim analysis (Nature Communications in press, 2023).

Outcomes

The primary safety objective was to evaluate the safety and reactogenicity of 50-µg mRNA-1273.222 when administered as a second booster dose. Safety assessments included solicited local and systemic adverse reactions ≤7 days and unsolicited adverse events ≤28 days post-booster administration, and serious adverse events, adverse events leading to discontinuation from study vaccine and/or participation, medically-attended adverse events, and adverse events of special interest from day 1 through the entire study period (~6 months).
The pre-specified primary immunogenicity objectives were to demonstrate non-inferior nAb or superior nAb responses against omicron BA.4/BA.5 and non-inferior nAb responses against ancestral SARS-CoV-2 with the D614G mutation (ancestral SARS-CoV-2 [D614G]), 28 days after the second booster dose (day 29) of mRNA 1273.222 50-µg compared with mRNA-1273 50-µg (Table S1 and statistical methods). Non-inferiority objectives were based on endpoints of geometric mean titer [GMT] ratio [GMR] and seroresponse rate [SRR] differences and those for superiority were based on GMR.

The incidence of symptomatic and asymptomatic SARS-CoV-2 infection was an exploratory objective.
